# Different Trajectories of Prolonged Grief in Bereaved Family Members After Terror

**DOI:** 10.3389/fpsyt.2020.545368

**Published:** 2020-10-14

**Authors:** Pål Kristensen, Kari Dyregrov, Rolf Gjestad

**Affiliations:** ^1^Center for Crisis Psychology, University of Bergen, Bergen, Norway; ^2^Faculty of Health and Social Sciences, Western Norway University of Applied Sciences, Bergen, Norway; ^3^Research Department, Division of Mental Health, Haukeland University Hospital, Bergen, Norway

**Keywords:** bereavement, terror, prolonged grief, trajectories, latent class growth analysis

## Abstract

**Introduction:** The loss of a loved one in a terror incident is associated with elevated risk for mental health disorders such as prolonged grief disorder (PGD) and posttraumatic stress disorder (PTSD), but the long- term adaptation after such losses are not well understood. This study aims to explore the trajectories of PGD among parents and siblings (*n* = 129) after the 2011 terror attack on Utøya Island, Norway.

**Methods:** The 19-item Inventory of Complicated grief (ICG) was used to measure PGD at 18, 28, and 40 months post-loss. Latent class growth analysis (LCGA) was used to identify trajectories of grief and a multinomial regression analysis was conducted to examine predictors of class membership.

**Results:** The analysis identified three grief trajectories; *moderate/decreasing* class (23%), *high/slow decreasing* class (64%), and a *high/chronic* class (13%). Predictors of high/slow recovery or chronic grief was female gender, previous depressive symptoms, and intrusion and avoidance symptoms.

**Conclusion:** The findings highlights the difficult grief process and slow recovery that characterizes the majority of close family members bereaved by a terror-incident. Community mental health programs should strive for both early outreach and long-term follow-up after such incidents.

## Introduction

Prolonged grief disorder (PGD) is a chronic, unrelenting grief that was included in the revised International Classification of Diseases (ICD-11) in 2018 (1) and is now also proposed for inclusion in DSM-5. PGD is distinguishable from other mental disorders such as depression and PTSD (2, 3), and is associated with poor physical health, reduced quality of life, and functional impairment (4). The core symptoms of PGD are intense yearning or preoccupation with the deceased, combined with severe emotional pain related to the loss (e.g., difficulties accepting the death, anger or bitterness, feeling that life is meaningless). PGD affects ~10% after expected losses, but certain subgroups show higher prevalence rates. A recent meta-analysis found that 50% of bereaved after unnatural losses (e.g., suicide, homicide, accident or combat-related deaths) experience PGD (5).

Persons who lose a loved one to terror-incidents are more vulnerable for developing PGD and other mental health disorders than persons bereaved by other types of disasters (5). High prevalence of PGD (43–83%) and comorbid PTSD (43–85%) have been found after different terror-incidents such as 9/11 (6), the 2011 Utøya-killings (7), and the 2015 terror attack in Paris (8). Long-term studies are limited, but one recent study by Cozza et al. (9) examined the mental health status of bereaved family members 15 years after the 9/11 incident and concluded that the majority of family members could be considered healthy.

Both degree of exposure to the incident and closeness in relationship to the deceased affects the level of mental health difficulties. In a study of 704 adults bereaved by the 9/11 terror attacks Neria, Gross et al. (6) found elevated levels of PGD among persons that witnessed the attacks on television and among parents who had lost a child.

The co-existence of trauma reactions or PTSD symptoms and grief may create a synergy effect that delays recovery (10). A common assumption is that traumatic imagery or intrusions may hinder normal grief from progressing. However, recent studies have also suggested that increase in grief levels predict later levels of PTSD and not vice versa (11, 12). Overall, the combination of PGD and PTSD is associated with functional impairment (13).

Longitudinal bereavement studies have traditionally relied on mean scores to measure grief reactions across time. While these studies are informative, mean scores do not capture the diversity of grief reactions over time (14). More advanced quantitative methods, latent class growth analysis are able to identify subpopulations or different pathways to adjustment, also termed trajectories. Few studies have investigated trajectories of grief or PGD after disasters. In a study of 170 adults bereaved by the South East-Asian tsunami in 2004 Sveen et al. (15) examined the course of PGD on three different occasions up to 6 years after the disaster. Three distinct trajectories were identified: recovery (48% of the sample), resilient (41%), and chronic (11%). The strongest predictor of chronic grief was loss of a child. Lenferinket al. (16) examined trajectories of PGD, depression, and PTSD on four occasions up to 2½ years post-loss among 172 adults bereaved by a plane disaster in Ukraine in 2014. Two trajectories of grief were identified: mild (82%) and chronic (18%). Low education was associated with the chronic grief trajectory. These studies demonstrate that the majority of disaster-bereaved adults will adjust to their loss, but a significant minority continue to struggle with chronic grief reactions several years after the death.

While studies of disaster-bereaved populations suggest that resilience and recovery are the two most common trajectories, no study has, as far as we know, examined trajectories of PGD among close family members after a terror-incident. Subsequently, our knowledge of their long-term adaptation after terror is scarce.

## Aim of the Study

The main aim of this study was to examine the trajectories of PGD among close family members bereaved by the 2011 terror attack at Utøya, Norway. A second aim was to investigate predictors of the different grief trajectories. We chose factors that has shown to predict PGD in other studies such as gender (17), type of loss (18), exposure to the incident (18), previous losses (19) and pre-loss mental health difficulties (20). In addition we chose to explore whether trauma reactions such as intrusions and avoidance would affect recovery from their loss (21). Satisfaction of social support was also included since it is regularly mentioned among the bereaved as important for recovery. These factors were all measured on timepoint 1.

## The Event

On July 22nd, 2011 a Norwegian born terrorist detonated a 950 kg car bomb close to several governmental buildings in the city of Oslo, killing 8 persons and severely injuring 10. Just before the bomb went off the terrorist drove to Utøya, a small island outside of Oslo, where over 500 adolescents and young adults were attending a political youth summer camp for the Norwegian Labor party. The terrorist, dressed as a police officer, chased people all over the island for 1 h and 20 min with the aim of killing as many as possible. Before he was arrested, he had killed 69 persons between 14 and 51 years of age, and physically injured 56.

## Materials and Methods

This project is derived from a longitudinal study, which examines the mental health effects of the loss of a child or sibling in the 2011 terror attack at Utøya Island, Norway. Participants completed a self-report questionnaire 18 (T1), 28 (T2), and 40 months (T3) after the attack.

## Procedures and participants

Biological parents and siblings, as well as step-parents and step-siblings of the adolescents and adults who were killed at Utøya were invited to participate in the study. Public records of the deceased were linked to the National Population Register (NPR) in order to obtain the names of the biological parents and siblings. The parents and siblings were first sent an informational letter regarding participation in the study. Those who wanted to participate returned their informed consent forms and received the questionnaire by ordinary mail or filled it in using Survey Monkey (a digital platform). Parents and/or siblings who were on Utøya during the terror attack were excluded from the study since they were invited to participate in a project on the direct survivors' mental health. As two of the deceased were non-Norwegian citizens and not registered in the NPR, their families were also excluded from the study. Moreover, one of the deceased had no parents alive.

A total of 208 biological parents and siblings were identified through the NPR, and 121 of them participated (58%). In addition, seven step-parents and one step-sibling participated in the study. Thus, the total sample consisted of 129 bereaved, that is, 86 parents and 43 siblings. At T1 103 persons participated, at T2 123 persons participated (including 25 new persons), and at T3 111 persons participated.

A total of 83 (64%) persons participated at all three time points. A total of 17 persons (13.2%) dropped-out from the study either after T1 (*n* = 5) or T2 (*n* = 12). There were no significant differences between ‘drop-outs’ and ‘completers’ neither on the sumscore on the ICG (M = 39.18 vs. M = 36.62), *t* = 0.75 (96), *p* = 0.46), on the intrusion subscale (M = 17.12 vs. M = 16.15, *t* = 0.51 (96), *p* = 0.61) nor on the avoidance subscale (M = 15.35 vs. M = 13.06, *t* = 1.28 (96), *p* = 0.20). The participants came from 51 families representing 74% of the deceased, with one-to-six members belonging to the same family.

The project was approved by the Regional Committees for Medical and Health Research Ethics in Norway.

## Measures

The survey consisted of questions mapping socio-demographics, loss-related questions, questions related to exposure, previous depressive symptoms, previous losses, psychosocial help after the terror-incident, PTSD and PGD.

Exposure was measured with the following question: “*Were you in contact with your child or sibling either by telephone or SMS during the terror attack”*? (yes vs. no). Previous depressive symptoms were measured with the following question: *Have you ever before July 22nd 2011 had low mood, felt depressed or had feelings of hopelessness for 4 weeks or more* (yes vs. no)? Previous losses were measured with the following question: *Have you any time before July 22nd 2011 lost someone through death whom you were closely attached to?* (yes vs. no). Received psychosocial help was measured with the following question: *Have you received help from mental health care after your loss?* (yes vs. no).

The *Inventory of Complicated Grief* (ICG) (22) was used to measure PGD. The ICG consists of 19 items; answers were ranked on a 5-point scale ranging from never (0) to always (4). Scores vary from 0 to 76 with a high score indicating a higher level of PG. The ICG has shown high internal consistency, test–retest reliability, and concurrent validity (22). The Norwegian version of ICG has shown good psychometric properties (23), and the internal reliability was found to be good across all three time points (α = 0.89–0.92). A cut-off score >25 indicates probable PGD (24).

The *Impact of Event Scale-Revised* (IES-R) (24) was used to measure symptoms of post-traumatic stress. IES-R consists of 22 items divided into three subscales: intrusion (re-experiencing the traumatic event), avoidance (avoiding trauma reminders), and hyperarousal (disturbed sleep, irritability, hypervigilance). Replies were given on a 5-point scale ranging from not at all (0) to extremely (4). The Norwegian version has shown good psychometric properties in a non-clinical sample (25). The internal reliability was found to be good across the three time points for intrusion (α = 0.83–0.90), for avoidance (α = 0.83–0.85), and arousal (α = 0.86–0.88).

One item from The *Crisis Support Scale* (26) was used to measure satisfaction with social support: “*Overall, are you satisfied with the social support you have received after the terror incident?”* This question has been used in other disaster studies (27). The respondents rated their overall satisfaction on a seven-point Likert scale, ranging from 1 (never) to 7 (always).

## Statistical Analysis

SPSS 23.0 was used for descriptive statistics, frequency, cross tabulation, student *t*-test, generalized mixed logistic regression, and linear mixed models (28). These two mixed (multilevel) models were used in order to account for family clustering in the data. The *p*-level was set to 0.05 (two-tailed). Mplus 7.4 was used for analyzing level and change in PGD as latent contrast difference score models (29). This model, which is a restricted latent growth curve model, analyzing each interval as separate pieces of change, was combined with mixture modeling in order to explore data heterogeneity and identify different sub-groups (latent classes) with different levels and change profiles of PGD (30). The mixture model was specified as a Latent Class Growth Analysis (LCGA) which constrain the variances in intercept and slope factors constrained to zero. This model may give one more class than the growth mixture model (GMM), with these variances estimated freely.

Based on an unconditional latent difference score in PGD, LCGA were analyzed (31). Evaluation of the number of classes was based on the entropy index, and the model fit indices from Akaike Information Criterion (AIC), the Bayesian Information Criterion (BIC) and sample-adjusted BIC (SA-BIC). Lower AIC, BIC, and SA-BIC values indicate a better model fit (32, 33), while entropy values should preferably be over 0.80, at least 0.70 (31). BIC and bootstrap LRT have been found to perform best out of all these indices across several simulated models (34).

Statistically significant improvement as a result of adding classes was tested by the Vuong-Lo-Mendell-Rubin Likelihood Ratio Test (LRT), and by a parametric bootstrapped likelihood ratio test for k-1(H0) vs. k classes with 1,000 bootstrap draws (35). The evaluation was in addition based on the estimated class size and relative class frequencies. A sample size of 25 classified subjects (5%) has been proposed (36).

The Maximum Likelihood Estimator with robust standard errors (MLR) was chosen in order to account for non-normality (32). Due to observation within families, the clustering option was used in order to achieve correct standard errors. The Full Information Maximization Likelihood method uses all available data under the “Missing at Random” assumption (32, 37). Thus, the effect of missing data is minimized (38), in contrast to listwise deletion methods, and ensures stronger statistical power and generalizability to the results. However, this do not rule out that missingness may be related to the values that would be observed if given, the “missing not at random” situation.

After the model with the best fit was determined, a multinominal logistic regression analysis examined potential predictors of class membership, including gender, type of loss, previous losses, previous depressive symptoms, exposure to the attack, received psychosocial help after the attack, social support (measured at T1), intrusion (measured at T1), and avoidance (measured at T1). The multinominal regression compares each group with a reference category.

## Results

The characteristics of the sample and descriptive statistics of PG is shown in Table 1. A total of 67% had lost a child among the participants. About half the parents were female (52.3%). Their mean age was 51.6 years (*SD* = 6.9), and almost all parents were married or cohabitants (85.5%). The mean age of the deceased children was 18.7 years (*SD* = 5.4). In the sibling sub-group 74.4% were female. Their mean age was 22.3 years (*SD* = 7.6), and 55.8% had lost a sister. The mean age of the deceased siblings was 18.6 years (*SD* = 5.2).

**Table 1:** Sample characteristics and mean sum scores on ICG and IES-R (*n* = 129).

	**Frequency**	**Percentage**
**Age**		
12–24	27	20.9
25–40	17	13.2
40+	85	65.9
**Gender**		
Female	77	59.7
Male	52	40.3
**Type of loss**		
Child	86	66.7
Sibling	43	33.3
**Exposed to incident**		
Yes	22	24.2
No	107	75.8
**Pre-22.7 depression**		
Yes	24	24.5
No	105	75.5
**Previous loss**		
Yes	100	77.5
No	29	22.5
**Psychosocial help after 22.7**		
Yes	88	68.2
No	41	31.8
	**Mean (range)**	**SD**
SS—satisfaction with social support (at T1)	4.67 (1–7)	1.59
IES-R Intrusion (sumscore at T1)	16.38 (1–32)	6.41
IES-R Avoidance (sumscore at T1)	14.36 (0–30)	6.34
ICG (total sumscore at T1)	37.52 (4–66)	11.59
ICG T2 (total sumscore at T1)	34.49 (2–72)	13.92
ICG T3 (total sumscore at T1)	32.46 (3–70)	13.48

**Figure 1:**
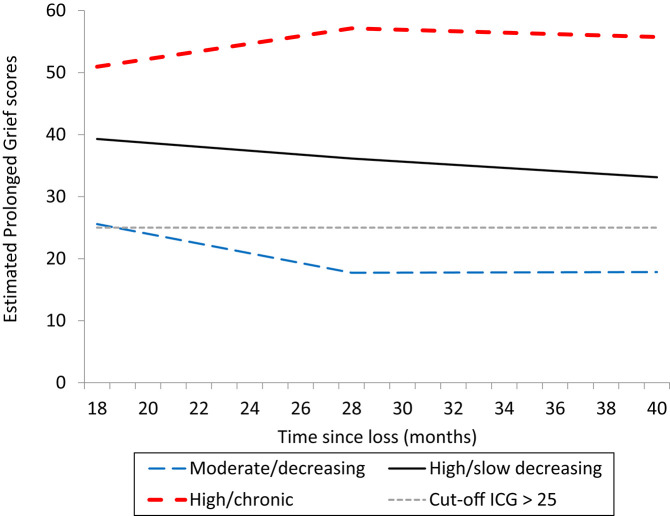
Different grief trajectories among bereaved family members after the 2011 terror attack on Utøya lsland.

Based on the fit measures and the number of subjects and frequencies, a three-class solution was decided to be the final model (Table 2). The six-class model represented the variability in the observed scores even better but could not be used for further analyses due to the very low sample size in several classes. The three-class model is presented in Figure 1. The subjects in the *moderate/decreasing class* (N = 29/22.5%) started with a PG score of 25.58, and showed a statistically significant reduction in the first period (−0.78, *p* < 0.01), but not in the second period (0.01, *p* = 0.972). The *high/slow decreasing class* (*N* = 83/64.3%) started with a PG score of 39.30 and showed statistically significant reduction in both time intervals (T1–T2 = −0.32, *p* < 0.01; T2–T3 = −0.25, *p* < 0.05). The *high/chronic grief class* (*N* = 17/13.2%) started with a PG score of 50.95, increased their scores significantly in the first interval (T1–T2 = 0.62, *p* < 0.05), but showed no further change (T2–T3 = −0.12, *p* = 0.526).

**Table 2:** Latent class growth analysis (LCGA) of the PG sum score with fit measures for evaluation (*n* = 129).

**Classes**	**AIC**	**BIC**	**SABIC**	**LRT**	**LRTs**	**Entropy**	**Least class**
				***p*-value^a^**	***p*-value^b^**		**predicted *n* (%)**
1	3,090	3,107	3,088	-	-	-	-
2	2,987	3,016	2,984	0.310	0.000	0.71	55 (42.6)
3	2,906	2,946	2,902	0.004	0.000	0.89	17 (13.2)
4	2,891	2,942	2,885	0.142	0.001	0.81	15 (11.6)
5	2,886	2,950	2,880	0.259	0.099	0.80	14 (10.9)
6	2,887	2,961	2,879	0.689	-	0.81	8 (6.2)

*AIC, akaike information criterion; BIC, bayes information criterion, SABIC, sample-adjusted bi; LRT, likelihood ratio test*.

a *Vuong-Lo-Mendell-Rubin LRT for k-1(H0) vs. k classes*.

b *Parametric bootstrapped LRT for k-1(H0) vs. k classes (1,000 bootstrap draws). Clustering corrections removed in order to being able to estimate the models*.

The generalized mixed multinomial regression results are presented in Table 3. Adjusted predictor estimates showed an increased probability for being in the *high/slow decreasing class* in contrast to the *moderate/decreasing class* (reference), if the subject was female, reported symptoms of depression before the attack, and had higher scores on intrusion (measured at T1). We found increased probability for being in the *high/chronic grief class* in contrast to the *moderate/decreasing grief class* (reference) if the subject was a female, reported symptoms of depression before the attack, and had higher scores on intrusion or avoidance (both measured at T1). The type of loss was not related to being in the *high/chronic grief class*.

**Table 3:** Trajectory group (class) prediction based on generalized mixed multinomial regression analyses.

**Class predictors**	**B**	***p***	**Exp (B)**	**95% CI**	**95% CI**
				**Low**	**High**
**High/decreasing**					
-**Gender**	1.88	**0.007**	6.57	1.66	25.99
-Loss (child)	0.16	0.823	1.17	0.29	4.77
-Exposure	0.35	0.662	1.42	0.29	6.88
-**Previous depressive symptoms**	3.03	**0.012**	20.67	1.96	218.33
-Previous loss	1.25	0.072	3.50	0.89	13.72
-Satisfied with social support (at T1)	−0.39	0.119	0.68	0.41	1.11
-Received psychological help (at T1)	−0.50	0.557	0.61	0.12	3.20
-**Intrusion (at T1)**	0.16	**0.010**	1.17	1.04	1.33
-Avoidance (at T1)	0.08	0.162	1.09	0.97	1.22
**High/chronic**					
-**Gender**	3.31	**0.005**	27.48	2.79	271.17
-Loss (child)	2.02	0.082	7.53	0.77	73.26
-Exposure	1.75	0.102	5.73	0.71	46.34
-**Previous depressive symptoms**	3.23	**0.025**	25.24	1.50	425.71
-Previous loss	0.31	0.762	1.36	0.19	9.92
-Satisfied with social support (at T1)	−0.47	0.165	0.62	0.32	1.21
-Received psychological help (at T1)	0.95	0.404	2.60	0.28	24.34
-**Intrusion (at T1)**	0.32	**0.001**	1.38	1.14	1.67
-**Avoidance (at T1)**	0.26	**0.009**	1.30	1.07	1.58

*The Moderate level/improved class was set as reference category. Intercept values not presented (n = 129)*.

## Discussion

This is, to our knowledge, the first study of trajectories of PGD following a terror-incident, and we found that a three-class model best represented the data. All three groups started above the recommended threshold for probable PGD (22), but had different developments across time. The classes included a high/slow recovery trajectory of (64%) with initial high level of grief which attenuated slowly from 1 to 3½ years after the incident; a moderate/decreasing trajectory (23%) with an initial moderate to high level of grief, which decreased below the threshold for PGD and a chronic trajectory (13%) which started with very high level of grief which even increased slightly from 1 to 3 1/2 years after the loss.

The lack of a a distinct resilient group is noteworthy, since most disaster-studies find resilience to be among, if not the most common trajectory (15, 16). Still, it is consistent with studies that suggest that resilience is far from normative among grieving parents (39). It is worth noting that the sample in the current study was homogenous constituting only of parents and siblings of those who were killed. Closeness in the relationship to the deceased is a stronger risk factor for PGD than both the circumstances of the death and time since the loss (40, 41). Also, almost all of those who were killed were adolescents between 14 and 19 years of age, which is an age that has been associated with more intense parental grief than loss of either a younger child or adult child (42). Also, some suggest that vulnerable persons find more meaning in participating in research (43). If this is true we may have a biased sample. On the other hand, those who struggle most with their grief may also refuse to participate in order to avoid reminders of the loss (44).

The finding that almost 80% of the participants displayed either a high grief level and a slow recovery or chronic grief is alarming, and suggest that the large majority of terror-bereaved family members may struggle for several years with adapting to their loss. Several factors may account for this. In addition to losing a child or a sibling, which is a devestating experience in itself, the terror-bereaved need to cope both with the traumatic circumstances of the loss and with secondary stressors such as media coverage and a potential trial which function as constant reminders of the incident. This affects their opportunity to grieve privately, but also to take time off from their grief (45).

Our finding that pre-loss depressive symptoms predicted chronic grief is regularly documented in studies of PGD, and should be routinly screened for after traumatic losses (4). Also, our finding that comorbid PTSD symptoms intrusion and avoidance predicted a slow recovery and chronic grief are consistent with a recent meta-analysis (21). Horowitz stated that intrusions are common after sudden traumatic events, and that avoidance is a strategy that is used to ward off the painful emotions related to these intrusions (46). While avoidance can be adaptive in the early stages of a loss, if it is used as the main coping strategy to regulate grief it may hinder the bereaved from confronting the reality of their loved one's death or processing the loss (47). Intrusive thoughts or re-enactment fantasies of how their loved one was killed can interfere with positive reminiscing of the deceased (48). This suggest that PTSD symptoms may hinder or delay the resolution of grief. Recent studies have, however, found that an increase in grief levels can predict later PTSD levels and not vice versa (11). This suggest that PGD and PTSD may influence each other reciprocally, which requests a thorough assessment in clinical practice since it may have implications for the choice of treatment.

## Limitations

The current study has several limitations. Due to confidentiality, the ethical committee did not permit inquiries about non-responders beyond the information of gender and age of the deceased. However, there were no statistically significant group differences between participants and non-participants concerning these demographic variables. Furthermore, the study suffer from low sample size. In the LCGA two classes had a relatively small number of predicted persons. The estimated relationships are a result of the included predictors, but also a result of the potential predictors not included in the model. Also, we used single non-validated questions in the assessment of previous depressive symptoms and psychosocial help after the attack. These measures may be subject to reporting bias. The use of ICG to tap grief symptoms does not capture the current symptoms or criteria for of PGD as defined in either DSM-5 or ICD-11 (1). Comparative studies show that it matters which criteria are used to define disturbed grief in terms of prevalence and predictive validity (49).

## Clinical Implications

There are several implications of these findings. First of all, the high level of PGD and slow recovery characterizing the majority of bereaved suggest that an early out-reach in the follow-up of family members bereaved by terror is warranted (50). Use of adequate screening measures may be helpful in reaching those who need help most. As studies have shown, persons with PGD may not seek adequate help due to different barriers, and not all will accept help in the early stages of the loss. A plan for long-time follow-up of the bereaved is also indicated. Some bereaved will need help first several months after their loss when there is a marked erosion of support from social network. Collective gatherings are highly valued among bereaved families were a combination of psychoeducation and grief groups are organized (51). Some bereaved also need specialized help for unrelenting PGD. Clinicians need to be familiar with assessment and treatment of both trauma-related disorders and different grief disorders. Several treatment models of traumatic bereavement are promising and have shown good effects (52, 53).

## Conclusion

Terror-bereaved family members are vulnerable for a long-lasting and difficult grief process, comprising of both trauma symptoms and PGD. This suggest that there is a need for both early interventions and interventions capturing those who need long-term help. Also, mental health personnel working with terror-bereaved families should have be trained in both assessment of and treatment of trauma or PTSD and PGD.

## Data Availability Statement

The raw data supporting the conclusions of this article will be made available by the authors, without undue reservation.

## Ethics Statement

The studies involving human participants were reviewed and approved by Regional Committees for Medical and Health Research Ethics in Norway. Written informed consent to participate in this study was provided by the participants' legal guardian/next of kin.

## Author Contributions

PK: lead author of introduction, materials and methods section, and discussion. KD: project leader, commented on manuscript. RG conducted analysis, written statistical analysis section, results section, commented on manuscript. All authors contributed to the article and approved the submitted version.

## Conflict of Interest

The authors declare that the research was conducted in the absence of any commercial or financial relationships that could be construed as a potential conflict of interest.
